# Effects of growth hormone on lipid metabolism and sexual development in pubertal obese male rats

**DOI:** 10.1515/biol-2022-0515

**Published:** 2022-11-24

**Authors:** Shujuan Guo, Juan Zheng, Guimei Li

**Affiliations:** Department of Pediatrics, Shandong Provincial Hospital, Cheeloo College of Medicine, Shandong University, Jinan, Shandong, 250021, China; Department of Pediatrics, Liaocheng People’s Hospital, Liaocheng, Shandong, 252000, China; Department of Joint Laboratory for Translational Medicine Research, Liaocheng People’s Hospital, Liaocheng, Shandong, China

**Keywords:** obesity, growth hormone, lipid metabolism, aromatase, testosterone

## Abstract

To investigate the effects of growth hormone (GH) on pubertal obese male rats, a rat model of high-fat diet-induced obesity was established in juvenile male rats. The model rats were divided into the treatment group (GH) and the non-treatment group (physiological saline). After 4 weeks, we measured the levels of alanine transaminase (ALT), aspartate aminotransferase (AST), total cholesterol (TC), triglycerides (TGs), high-density lipoprotein cholesterol (HDL-C), low-density lipoprotein cholesterol (LDL-C), estrogen (E2), testosterone (T), and insulin-like growth factor (IGF-1). The morphological changes of the liver and testis were assessed, and the expression of aromatase was detected. The levels of ALT, AST, TC, TG, LDL-C, E2, and IGF-1 in the treatment group were significantly lower than in the non-treated model rats (*P* < 0.001). The levels of HDL-C and T of GH-treated rats were significantly higher than those of the non-treatment group (*P* < 0.001). Compared with non-treated model rats, GH-treated model rats showed reduced liver steatosis, improved morphological structure of the testicular seminiferous tubules, and an increased number of spermatogenic cells. The treatment group also showed lower expression of aromatase in the liver and testis compared with the non-treatment group. GH partially protected pubertal male rats from obesity-induced lipid metabolic disorder and sexual retardation.

## 1 Introduction

Obesity is a disorder of energy balance characterized by the accumulation of excessive body fat. Specific lifestyle behaviors, such as excessive intake of high-fat and high-calorie foods and a lack of exercise, have led to an increased prevalence of obesity in children and adolescents. In the past decade, the prevalence of childhood obesity in the United States has continued to increase, and approximately one-fifth of children were obese in 2016 [1]. The rate of obesity in Chinese boys has increased from 9 to 15.4% between 2007 and 2016 and now ranks seventh in the world [2]. Obesity has been shown to affect lipid metabolism and increase the risk of metabolic syndrome [3]. Obesity also reduces testosterone levels, resulting in male secondary hypogonadism [4,5]. The incidence of obesity and sexual retardation has been increasing in male children, and how to properly treat these children has become an important clinical issue.

Growth hormone (GH), a protein hormone secreted by the adenohypophysis, stimulates tissue growth through anabolism. GH has currently been used in many clinical fields, such as short stature in children [6], GH deficiency in adults [7], and burn injury [8]. Previous studies have shown that GH not only induces lipolysis and improves lipid metabolism [9] but also improves the function of Leydig cells and sperm quality, thus playing an important role in the reproductive system [10]. However, few studies have reported the effects of GH on obese male adolescents with dyslipidemia and delayed sexual maturity. In this study, we investigated the effects of GH on lipid metabolism and sexual development in pubertal obese male rats with the aim of providing scientific support for the clinical treatment of delayed sexual maturity in obese male adolescents.

## 2 Materials and methods

### 2.1 Reagents

Recombinant human GH was purchased from GenSci (Changchun, China). Paraformaldehyde was obtained from Shanghai Shenggong (Shanghai, China). Phosphate buffered saline (PBS) powder was purchased from Zhongshan (Beijing, China). Anhydrous ethanol was obtained from the Pharmaceutical Group (Beijing, China). Hematoxylin staining solution was purchased from Jiancheng (Nanjing, China). Eosin staining solution was purchased from Biyuntian (Shanghai, China). Neutral resin was obtained from the Sinopharm Group (Beijing, China). The primary antibody against cytochrome P450 (rabbit origin) was purchased from Zhengneng (Chengdu, China). An horseradish peroxidase (HRP)-labeled secondary antibody (goat rabbit) was obtained from Abcam (Burlingame, CA, USA).

## 3 Establishment of the rat model of obesity

Specific pathogen-free grade male standard deviation (SD) rats (3 weeks old, weighing 50–55 g, *n* = 41) were purchased from Beijing Huafukang Biotechnology Co., Ltd, Beijing, China. All animals were housed in an environment with a temperature of 23–25°C and had free access to food. Rats assigned to the model group (*n* = 26) were fed a high-fat diet, while those in the control group (*n* = 15) were fed regular chow. At the age of 7 weeks, the body length, body weight, waist circumference, penis length, and testicular size of all rats were measured. Lee’s index was calculated using the following formula: Lee’s index = \sqrt[3]{\text{body}\hspace{.25em}\text{weight}}] (g) × 1,000/body length (cm) [11].

The model was considered to be successfully established when Lee’s index was above the maximal value of the control group (mean ± SD) [12].


**Ethical approval:** The research related to animal use has complied with all the relevant national regulations and institutional policies for the care and use of animals and has been approved by the Ethics Committee of Liaocheng People’s Hospital (LPH-2019052).

## 4 Groups and treatment

At the age of 7 weeks, eight model rats were randomly divided into two groups (*n* = 4 per group): the treatment group and the non-treatment group. The treatment group received a daily subcutaneous injection of GH (3.0 IU/kg per day) for 4 weeks. The non-treatment group was subcutaneously injected with physiological saline by the same procedure. A control group of rats (regular chow-fed rats, *n* = 5) received a subcutaneous injection of physiological saline. All rats were fed regular chow during the treatment.

## 5 Measurements

Physical parameters, including body length, body weight, waist circumference, penis length, and testicular size, were monitored before dissection.

At 4 weeks following treatment, rats were anesthetized with 7% chloral hydrate (0.5 mL/100 g), and 10 mL of blood was collected via the abdominal aorta into a tube containing ethylene diamine tetra-acetic acid. After 2 h, the serum was collected and stored at −20°C. The levels of estrogen (E2), testosterone (T), and insulin-like growth factor (IGF-1) were measured using enzyme linked immunosorbent assay (ELISA) kits purchased from Beijing Likechuangxin Biotechnology Corporation (Beijing, China). The levels of alanine transaminase (ALT), aspartate aminotransferase (AST), total cholesterol (TC), triglycerides (TG), high-density lipoprotein cholesterol (HDL-C), and low-density lipoprotein cholesterol (LDL-C) were detected using ELISA kits obtained from the Nanjing Jiangcheng Bioengineering Institute (Nanjing, China).

## 6 Histopathological analysis

After blood collection from rats, the liver and testis were removed, washed with PBS, and fixed with 4% paraformaldehyde. The tissue was dehydrated, paraffin-embedded, and cut into 5-μm-thick sections. The sections were dewaxed to water and stained with H&E. The morphological changes of the liver and testis were observed under an optical microscope (400-fold magnification).

For immunohistochemical staining, tissue sections were dewaxed, blocked with goat serum, and incubated with primary antibody against aromatase (dilution ratio 1:100) overnight at 4°C. The sections were then incubated with HRP-labeled goat anti-rabbit antibody (dilution ratio: 1:50) at 37°C for 30 min. The sections were then observed under an optical microscope. The cytoplasm of the positively stained cells was yellow-brown. The integrated and average optical density of each slide was measured by Image-Pro Plus.

## 7 Statistical analysis

The data were analyzed using SPSS 23.0 software. The data that were normally distributed were expressed as mean ± standard deviation and compared using one-way analysis of variance followed by pairwise comparison using the least significant difference method. The skewed data were expressed as median (minimum, maximum) and compared by nonparametric test followed by pairwise comparison using the Kruskal-Wallis H rank-sum test. *P* < 0.05 indicated statistical significance.

## 8 Results

### 8.1 Comparison of physical parameters between model and control rats

To investigate the effects of GH on lipid metabolism and sexual development in pubertal obese male rats, we established a rat model of obesity. At 7 weeks of age, eight rats in each group were screened to determine whether the model was successfully established. The model group showed a significantly higher weight and the ratio of waist circumference to body length (W/H) compared with control rats (*P* < 0.05). The Lee’s index of the model group was also significantly higher than that of the control group (*P* < 0.05), indicating that the obesity model was successfully established. The penis length of model rats was significantly shorter than that of control animals (*P* < 0.05). However, no significant difference in the testicular volume was observed between the two groups (*P* > 0.05) (Table 1).

**Table 1:** Comparison of physical parameters of model and control rats

	Model rats (*n* = 8)	Control rats (*n* = 8)	*P*-value
Body weight (g)	194.43 ± 15.48	175.74 ± 13.09	0.021
W/H	0.79 ± 0.03	0.63 ± 0.03	<0.001
Lee’s index	315.08 ± 3.91	281.53 ± 5.15	<0.001
Penis length (mm)	8.99 (8.48, 10.04)	10.27 (9.78, 12.74)	0.002
Testicular volume (cm^3^)	1.84 ± 0.26	2.08 ± 0.37	0.171

### 8.2 Comparison of physical parameters among control, non-GH-treated model, and GH-treated model groups

At 4 weeks after treatment, GH-treated rats showed significantly lower Lee’s index and W/H and increased penis length and testicular volume compared with the non-treatment group (*P* < 0.05). There was no significant difference in body weight between the treatment and non-treatment groups (*P* > 0.05). No significant differences were observed in Lee’s index, penis length, and testicular volume between the treatment group and the control animals (*P* > 0.05). However, the body weight and W/H of GH-treated rats were significantly higher than those of the control group (*P* < 0.05) (Table 2).

**Table 2:** Comparison of physical parameters among control, non-GH-treated model, and GH-treated model groups

	Control group (*n* = 5)	Non-treatment group (*n* = 4)	Treatment group (*n* = 4)
Body weight (g)	272.26 ± 21.57	328.85 ± 16.44*	330.43 ± 25.95*
W/H	0.69 ± 0.03	0.87 ± 0.04*	0.78 ± 0.01*^
Lee’s index	292.26 (278.79, 294.69)	315.59 (300.79, 331.34)*	286.10 (284.33, 287.90)^
Penis length (mm)	11.96 (10.46, 12.82)	9.61 (9.06, 10.06)*	11.60 (11.44, 11.82)^
Testicular volume (cm^3^)	3.50 ± 0.23	2.56 ± 0.19*	3.62 ± 0.24^

*P < 0.05, compared with the control group; ^*P* < 0.05, compared with the non-treatment group.

### 8.3 Comparison of serological indices among control, non-GH-treated model, and GH-treated model groups

The ALT, AST, TC, TG, LDL-C, E2, and IGF-1 levels were significantly lower in the treatment group, while the levels of HDL-C and T were significantly higher in the treatment group compared with levels in the non-treatment group (*P* < 0.05). GH-treated model rats also showed significantly higher levels of ALT, AST, TC, TG, LDL-C, E2, and IGF-1, but lower levels of HDL-C and T compared with levels in the control group (*P* < 0.05) (Table 3).

**Table 3:** Comparison of serological indices among control, non-GH-treated model, and GH-treated model groups

	Control group (*n* = 3)	Non-treatment group (*n* = 3)	Treatment group (*n* = 3)
ALT (IU/L)	33.80 ± 1.33	153.09 ± 1.75*	82.66 ± 1.16*^
AST (IU/L)	27.47 ± 0.85	120.42 ± 0.63*	53.71 ± 2.09*^
TC (mmol/L)	6.36 ± 0.10	28.70 ± 0.29*	14.80 ± 0.37*^
TG (mmol/L)	2.57 ± 0.09	10.80 ± 0.14*	5.86 ± 0.06*^
HDL-C (mmol/L)	2.76 ± 0.22	0.81 ± 0.04*	1.88 ± 0.02*^
LDL-C (mmol/L)	2.93 ± 0.06	9.00 ± 0.04*	4.90 ± 0.03*^
E2 (ng/L)	5.14 ± 0.08	20.81 ± 0.63*	10.12 ± 0.23*^
T (ng/mL)	1.26 ± 0.01	0.22 ± 0.02*	0.87 ± 0.02*^
IGF-1 (μg/L)	5.11 ± 0.10	12.45 ± 0.47*	7.30 ± 0.22*^

*P < 0.05, compared with the control group; ^*P* < 0.05, compared with the non-treatment group.

### 8.4 Comparison of H&E staining of liver and testis among control, non-GH-treated model, and GH-treated model groups

**Figure 1:**
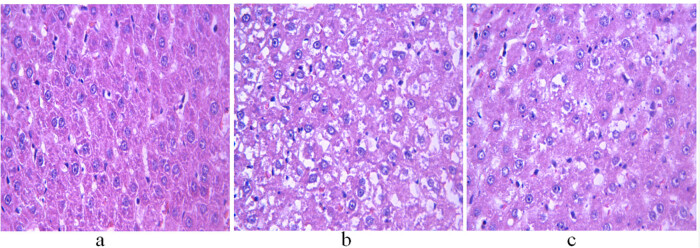
H&E staining of the liver of 11-week-old rats. (a) Control group; (b) non-treatment group; and (c) treatment group. Scale bar = 400 μm.

H&E staining showed that the hepatocytes of control rats had normal morphological structure, abundant cytoplasm, and large and round nuclei located in the center of the cells (Figure 1a). The non-treatment group showed severe hepatic steatosis, with lipid droplets of different sizes in the cytoplasm and nuclei squeezed to one side (Figure 1b). However, the hepatocytes of GH-treated rats showed reduced fat vacuoles, improved morphological features, and better arrangement compared with non-treated animals (Figure 1c).

**Figure 2:**
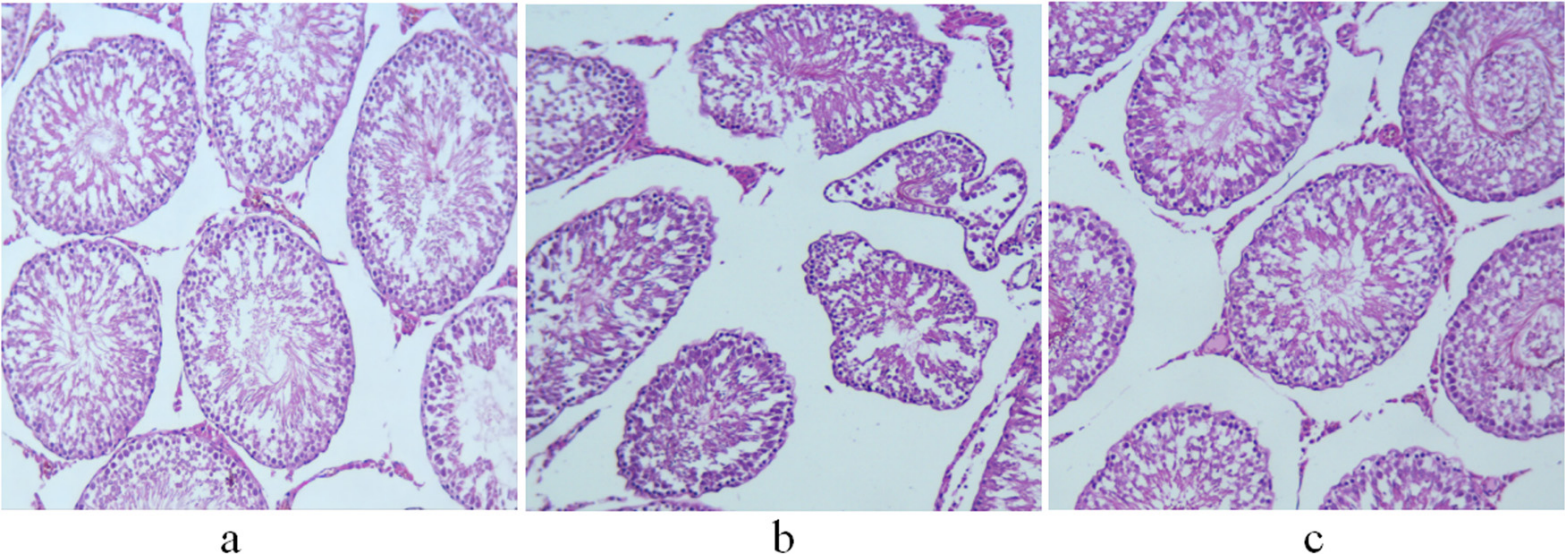
H&E staining of the testis of 11-week-old rats. (a) Control group; (b) non-treatment group; and (c) treatment group. Scale bar = 100 μm.

The seminiferous tubules of the control group had normal morphological structures. Spermatogenic cells were observed in the seminiferous epithelium. Spermatogenesis was observed near the lumen. Leydig cells were distributed singly or in groups between seminiferous tubules (Figure 2a). The non-treatment group showed atrophic seminiferous tubules and a reduced number of spermatogenic cells and Leydig cells. Some spermatogenic cells were necrotized and fell off, blocking the lumen (Figure 2b). Treatment with GH restored the morphological structure of the seminiferous tubules to a certain extent and increased the number of spermatogenic cells. In addition, the regeneration of spermatozoa was observed near the lumen, and Leydig cells were orderly distributed between seminiferous tubules (Figure 2c).

### 8.5 Comparison of immunohistochemical staining of aromatase in the liver and testis among control, non-GH-treated model, and GH-treated model groups

**Figure 3:**
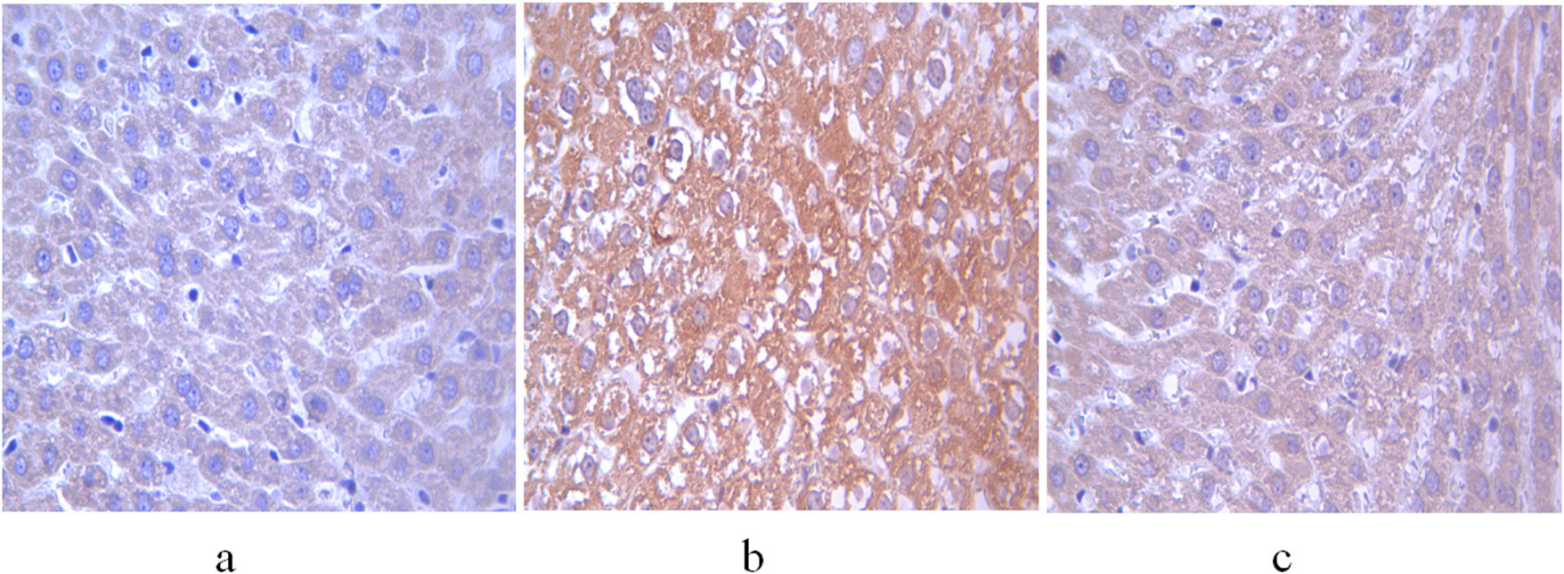
Expression of aromatase in the liver of 11-week-old rats. (a) Control group; (b) non-treatment group; and (c) treatment group. Scale bar = 400 μm.

**Figure 4:**
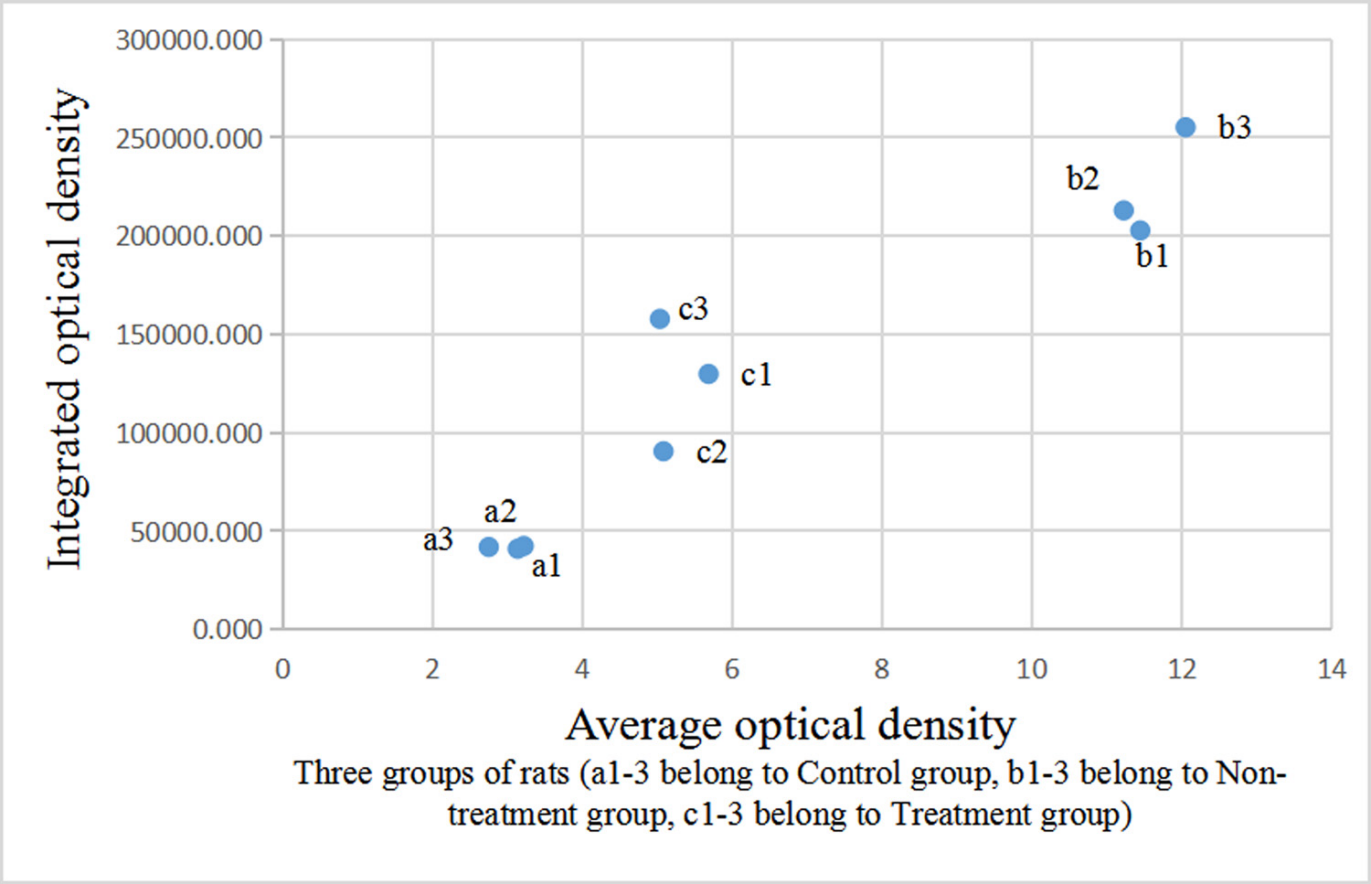
Comparison of optical density of aromatase-positive staining in the liver.

The integrated and average optical densities of aromatase-positive staining in the liver were significantly different among the three groups (*P* < 0.05). The integrated and average optical densities of the treatment group were increased compared with those of the controls (*P* < 0.05) but were significantly decreased compared with those of the non-treatment group (*P* < 0.05) (Figures 3 and 4).

**Figure 5:**
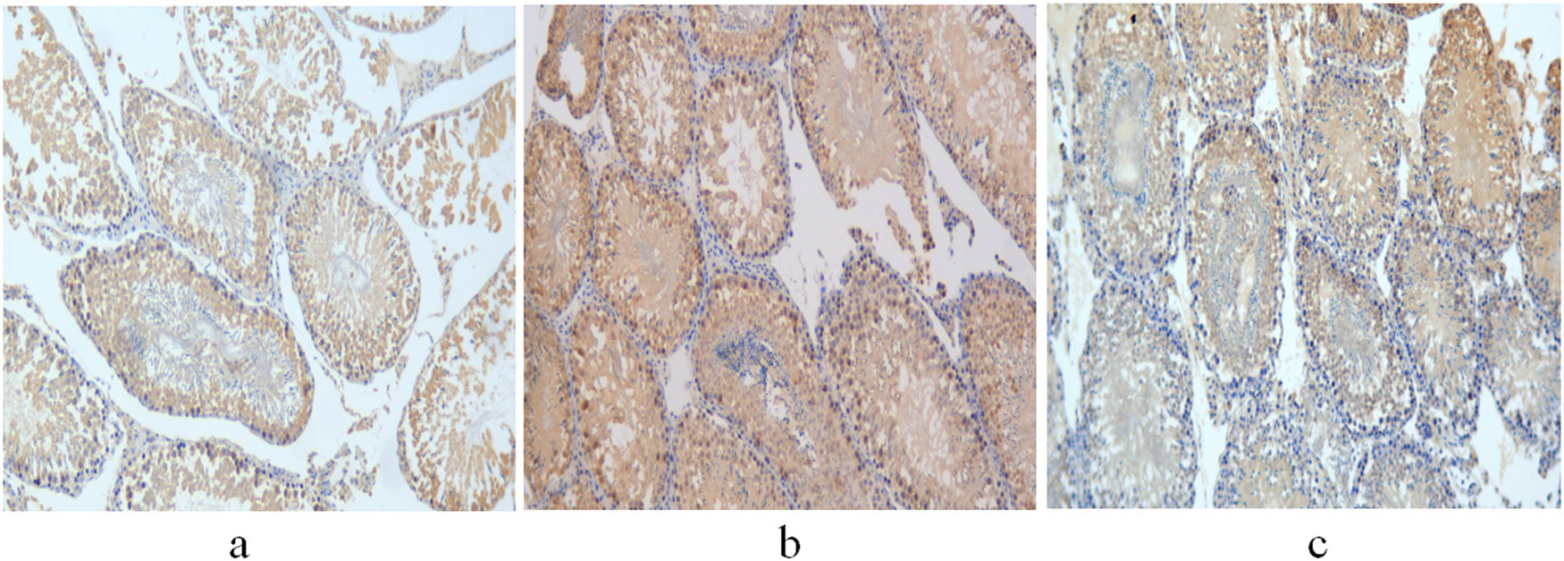
Expression of aromatase in the testis of 11-week-old rats. (a) Control group; (b) non-treatment group; and (c) treatment group. Scale bar = 100 μm.

**Figure 6:**
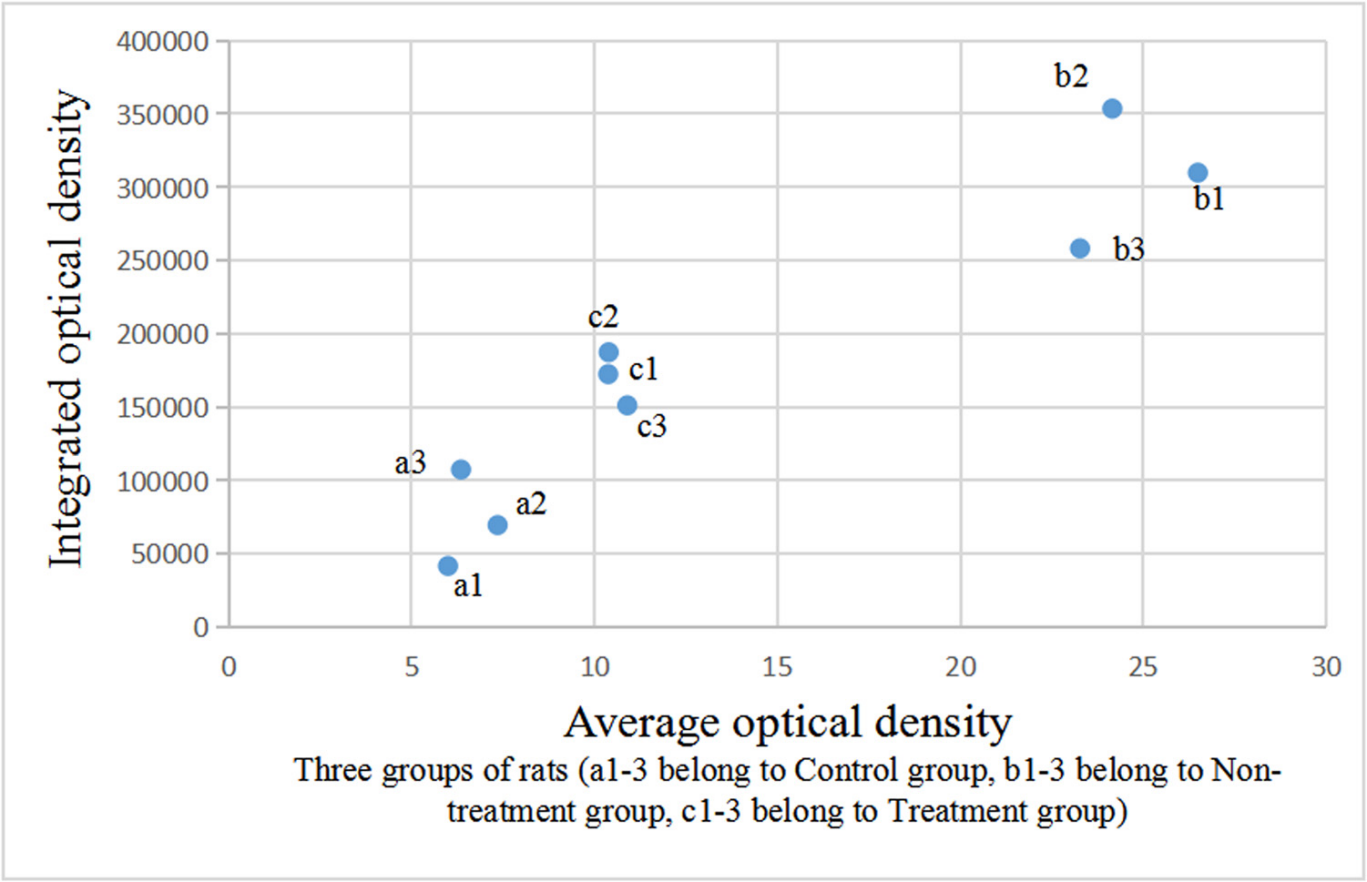
Comparison of optical density of aromatase-positive staining in the testis.

The integrated and average optical densities of aromatase-positive staining in the testis were also significantly different among the three groups (*P* < 0.05). The integrated and average optical densities of the treatment group were increased compared with those of the controls (*P* < 0.05) but were significantly decreased compared with those of the non-treatment group (*P* < 0.05) (Figures 5 and 6).

## 9 Discussion

A high-fat diet leads to an excessive accumulation of fat in the body. The mesenteric adipose tissues have the highest adipogenic activity; therefore, the continuous proliferation of abdominal adipocytes is a key contributor to abdominal obesity [13]. The W/H ratio is an important indicator of abdominal obesity [14]; a higher W/H value corresponds with a higher risk of metabolic disorders [15]. In this study, the body weight and W/H ratio of model rats were significantly higher than those of the control group at the age of 7 weeks, indicating that a high-fat diet before puberty led to abdominal obesity in rats. Continuous accumulation of fat increases the levels of circulating free fatty acids, contributing to the occurrence of hyperlipidemia, type 2 diabetes, and cardiovascular diseases [16]. The incidence of dyslipidemia in obese children is 28–46% [17]. Lipid spectrum disorders are usually manifested by increased TC, TG, and LDL and decreased HDL-C [17], which was in line with the blood lipid profiles of rats in the non-treatment group. In addition, excessive deposition of fat (mainly in the form of TG) in the liver may lead to adipokine imbalance, dysregulated apoptosis of hepatocytes, and enterogenic endotoxin invasion, resulting in nonalcoholic fatty liver disease and accompanied progressive liver damage [18]. At the age of 11 weeks, the non-treatment group showed significantly impaired liver function, with structural disorder and hepatocyte steatosis, suggesting that dietary management alone could not completely improve metabolic disorders in obese rats.

GH participates in lipid metabolism by binding to receptors on the surface of target cells; its receptors exist in a variety of tissues, but most receptors originate from the liver [19]. GH binding to its receptor results in the activation of multiple signaling pathways, leading to the regulation of the transcription of GH-responsive genes in the liver, and the interaction among these pathways has been shown to inhibit lipid synthesis and reduce the accumulation of TG [20]. GH also reduces the amount of fat by affecting the differentiation and proliferation of preadipocytes [21]. GH has a robust lipolytic effect on abdominal fat. Our results showed that the W/H of the treatment group was significantly decreased compared with that of the non-treatment group, which also confirms the above view. A previous study showed that GH treatment reduced visceral fat in obese adults without changing their body weight [21]. In our study, no significant difference in body weight was observed between the GH treatment and non-treatment groups. However, GH-treated rats showed a significantly decreased Lee’s index compared with the non-treatment group, which may be due to the linear growth-promoting effect of GH. Our results showed that the levels of TC, TG, and LDL-C were significantly lower in GH-treated rats, while the levels of HDL-C were higher than those in the non-treatment group. Evaluation by light microscope revealed that the hepatocytes in GH-treated rats were arranged in a more orderly way, the degree of steatosis was reduced, and the morphology and structure were restored to a certain extent. Although there were still differences compared with the control group, these findings indicated that dysregulated lipid metabolism in pubertal obese rats was ameliorated by GH treatment. With the decrease in lipid accumulation, the impaired liver function gradually recovered, which was directly manifested as the decrease of ALT and AST levels.

As the major anabolic mediator of the GH effect, IGF-1 depends on GH and regulates the secretion of GH through a negative feedback system [22]. Although IGF-1 is not involved in GH-induced lipolysis due to the absence of functional IGF-1 receptors in adipocytes [23], IGF-1 indirectly regulates lipolysis by affecting the structure and function of adipose tissues [20]. Our results showed that the levels of IGF-1 in model animals were higher than those in the control group; this may be because the stability and biological activity of IGF-1 are regulated by insulin-like growth factor binding protein-1 (IGFBP-1), a specific binding protein produced by the liver, as well as insulin *in vivo*. Adipose tissue as an endocrine organ is closely related to insulin sensitivity. Visceral fat accumulation induces the secretion of pro-inflammatory cytokines, including IL-6, TNF-α, and IL-8, which reduces insulin sensitivity and leads to hyperinsulinemia and even insulin resistance [24]. Insulin levels were not detected in this study, but we speculate that obese rats might have elevated insulin levels compared with controls. Hyperinsulinemia not only reduces the levels of IGFBP-1 [22] but also induces the production of IGF-1. Low levels of IGFBP-1 enhance the activity of IGF-1 [25], eventually leading to the upregulation of IGF-1 and the feedback inhibition of GH. At the end of our experiment, the levels of IGF-1 in the treatment group were significantly lower than those in the non-treatment group, which may be explained by decreased insulin levels caused by the reduction of abdominal fat.

Puberty is mainly characterized by sexual maturity and accelerated growth, both of which are related to the activation of the hypothalamic–pituitary–gonadal (HPG) axis and its coordination with the GH-IGF-1 axis [26]. Obesity-induced hypogonadism in males is related to excessive expression of aromatase, a complex cytochrome P450 enzyme that catalyzes the conversion of testosterone into E2 in adipose tissues [27]. An accumulation of adipose tissue enhances the function of aromatase, resulting in the downregulation of testosterone and upregulation of E2. Elevated E2 levels inhibit the activity of the HPG axis, which further impedes the production of testosterone [28]. Consistent with these findings, the non-treatment group in our study showed significantly increased expression of aromatase, decreased testosterone levels, and increased E2 levels compared with the control group. Obesity inhibits the secretion of GH *in vivo* [29]. GH deficiency not only affects the activity of testosterone and its derivative dihydrotestosterone but also reduces the number of androgen receptors in the prepuce [30], which affects the development of the penis. In our study, the penis length of 7-week-old model rats was significantly smaller than that of the control group, but the difference in the testicular volume was not significant, which may be related to the slow development of the testis before puberty.

GH is essential for the maturation of the mammalian reproductive system because it not only regulates the production of steroids but it also plays an important role in spermatogenesis and gonadotropin secretion. GH binds to receptors in Leydig cells and promotes testosterone synthesis by inducing the transcription of the steroidogenic acute regulatory protein and 3β-hydroxysteroid dehydrogenase genes, while testosterone regulates spermatogenesis by acting synergistically with follicle-stimulating hormone on Sertoli cells [31]. Experimental studies showed that GH treatment significantly increased sperm activity in infertile men [32] and improved the number and morphology of germ cells [33]. In our study, the expressions of aromatase in the liver and testis of the treatment group were lower than those of the non-treatment group. The secretion of testosterone in GH-treated rats was increased, which promoted the development of the reproductive system. There was no difference in penis length and testis volume between the treatment and control groups. Light microscopy showed the improved morphological structure of testicular seminiferous tubules and spermatogenesis in the treatment group, indicating that GH improves testicular function in model rats. The effects of GH on the growth of the testis and penis are mostly indirectly mediated by IGF-1. GH induces the secretion of IGF-1 by Sertoli cells, which functions synergistically with luteinizing hormone to regulate the production of androgen by Leydig cells, thereby promoting the development of the reproductive system [31].

The clinical application of GH has been shown to improve adolescent abdominal obesity and lipid levels. Kamel et al. treated six severely obese boys (10–12 years old) with GH for 6  without dietary adjustment and found that the overall percentage of fat was decreased and glucose homeostasis was not negatively affected [34]. Another study treated 18 obese boys (8–16 years old) with GH treatment for 1 year and found that their body mass index standard deviation scores and the levels of LDL-C, TG, TC, and alanine aminotransferase were decreased [35]. In addition, 21 obese women (13–21 years old) were reported to have decreased TC levels after 6 months of GH treatment, and the changes in IGF-1 levels were negatively correlated with those in TC, TG, and very low-density lipoprotein [36].

In this study, the dosage of GH and the route of exposure were chosen based on previous studies in male rats. Oh et al. established a micropenis rat model and found that treatment with GH (2.5 mg/kg, alternate days) promoted the growth of the penis and testis, and maintained structural integrity [30]. Huh et al. treated prepubertal rats with different doses of GH (1 or 2 IU/kg per day) and found that GH promoted the synthesis of testosterone and induced the early onset of puberty in a dose-dependent manner [37]. Administration of peripubertal rats with GH at a dosage of 2–11 IU/kg per day also promoted their body growth [37]. Few studies have tested the toxicity of GH at a high dose, which is worthy of further study.

Puberty in male rats is from 6–10 weeks after birth [38]. In this study, we established a model of obesity in juvenile male rats at 7 weeks of age. GH treatment partially protected pubertal male rats from an obesity-induced metabolic disorder and sexual retardation. Some serological indices of GH-treated rats were still significantly different from those of the control group. Whether a longer treatment duration or the combination with other treatments, such as exercise intervention, would exert better therapeutic effects warrants further investigation.

## 10 Conclusion

Our study provides scientific support for the use of GH in treating lipid metabolism and delayed sexual maturity in pubertal obese male rats. The clinical use of GH warrants further exploration.
